# Impact of Climate Change, Agricultural Credit and Inflation on Cereal Crop Productivity in Ethiopia: Novel Dynamic Simulated ARDL Approach

**DOI:** 10.1002/fsn3.71559

**Published:** 2026-02-18

**Authors:** Jianmin Cao, Gizachew Wosene, Yuhan Pang, Mezgebu Aynalem, Arshad Ullah Jadoon

**Affiliations:** ^1^ College of Economics and Management Jilin Agricultural University Changchun China; ^2^ Debre Markos University Bure Campus Department of Agribusiness and Value Chain Management Bure Ethiopia

**Keywords:** agricultural credit, climate change, Ethiopia, inflation, NDS‐ARDL

## Abstract

This study examines the impact of climate change, agricultural credit, and inflation on cereal crop productivity (CCP) in Ethiopia, using time series data from 1992 to 2022. Novel Dynamic Simulated Autoregressive Distributed Lag (NDS‐ARDL) model was applied for the empirical analysis. To address the dynamic effects, impulse response functions were simulated, indicating the impact of ±10% shocks for each independent variable on CCP. The bound test results show that the variable illustrates long‐term relationships. The coefficient of error correction term is −0.67, suggesting about 67% annual adjustment towards long run equilibrium. In the long‐run, fertilizer application, cropland, and agricultural subsidy showed positive and significant contributions, while CO_2_ and inflation showed a negative and significant impact on CCP. Furthermore, in the short‐run, agricultural credit has a positive and significant, while inflation showed a significant negative impact on CCP. To boost long‐term agricultural productivity, government should promote use of location‐specific quality fertilizers, improved land use policy, and sustain agricultural subsidies. Additionally, financial institution and agricultural cooperatives should provide affordable credit services for farmers to support short‐term productivity gains. Furthermore, to combat the adverse impact of CO_2_ emissions and inflation, government should promote climate‐smart agricultural practices and implement a price control policy on essential agricultural inputs.

## Graphical abstract

This study investigates the effect of climate change, agricultural credit, and inflation on cereal crop productivity in Ethiopia using time series data from 1992 to 2022. Novel Dynamic Simulated Autoregressive Distributed Lag (NDS‐ARDL) model was applied for empirical analysis. The results show that CO_2_ emission, fertilizer consumption, cropland, inflation, and agricultural subsidies have a long‐run relationship with crop productivity. In the short run, both inflation and agricultural credit exhibit a significant impact on crop productivity.

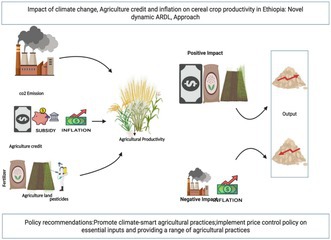

**Abbreviations**
CCP: cereal crop productivity
CRGE: climate‐resilient green economy
CSA: central statistical agency
ECT: error correction term
FAO: Food and Agriculture Organization
GDP: gross domestic product
IPCC: intergovernmental panel on climate change
NBE: National Bank of Ethiopia
NDS‐ARDL: novel dynamic simulated autoregressive distributed lag
SSA: Sub‐Sahara Africa
WDIs: world development indicators

## 1 Introduction

Agriculture plays a vital role in Ethiopia's socio‐economic development, accounting for about 34% of national GDP, generating 85% of the export earnings and serving as a source of livelihood for a major population (Eshete et al. 2020; NBE 2021). Despite its importance, the sector's productivity is constrained by numerous challenges, including climate change, limited access to agricultural credit, inflation and limited access to improved agricultural inputs (Gebeyehu and Bedemo 2024). Agricultural productivity is the rate of increase in total output per unit of input, either by producing more output with the same input or by maintaining the same output while using fewer inputs (Benton and Bailey 2019). It can be achieved through the use of improved inputs, management practices, and infrastructure, as well as access to credit. Improving agricultural productivity plays a vital role in meeting the growing demand for food, poverty reduction and improving the livelihood of rural households (Abdul‐Rahaman et al. 2021).

Cereal crops are the predominant agricultural activity in Ethiopia and serve as a vital source of livelihood for smallholder farmers (Chamberlin and Schmidt 2012; Taffesse et al. 2012). It also plays a crucial role in attaining food sovereignty, as they constitute the primary staple foods for most of the population and account for roughly 70% of the country's average calorie intake (Berhane et al. 2011). In 2019, cereals accounted for 87.97% of total grain production, with maize, tef, wheat and sorghum contributing 30.08%, 17.12%, 15.33% and 15.92%, respectively (CSA 2019).

Ethiopia's government has implemented major policy reforms in cereal production, leading to a substantial rise in production from 61.5 million quintals in 1994/95 to 269.7 million quintals in 2019/20, with an average growth rate of 6.6% (Birhanu et al. 2022). However, despite this notable increase, its productivity remains below its potential. The key contributing factor limiting the productivity of cereal crops is restricted access, limited use and improper use of production input; the adversity of weather variability and climate change (Amenu 2017; Urgessa 2016; Yu et al. 2011).

Climate change is one of the most pressing challenges undermining agricultural productivity in low‐income and climate‐vulnerable countries such as Ethiopia (Lechamo Gemecho et al. 2019; Manono et al. 2025; Tadesse and Barry 2024). Key drivers of climate change include carbon dioxide emissions, changes in annual mean temperature and rainfall, and forest depletion, which serve as useful proxies for measuring the long‐term environmental impact on agricultural productivity (Adinew and Gebresilasie 2019). It causes rising temperatures, shifting rainfall patterns, and extreme weather events, which strongly influence cereal crops in Ethiopia, such as teff, maize, wheat, millet, rice, and sorghum, which secure food sovereignty and the livelihoods of millions of rural households (Adinew and Gebresilasie 2019).

According to the Intergovernmental Panel on Climate Change (IPCC 2022) and Food and Agriculture Organization (FAO 2024), climate‐induced stress would affect crop productivity if climate‐smart agricultural practices are not implemented in Sab‐Sahara Africa (SSA). In Ethiopia, studies show that agricultural activities are prone to changes in climate and variation in the level of precipitation, posing threats to the food security of rural households (Bouteska et al. 2024).

Furthermore, credit plays a tremendous role in making agriculture modern and increasing farmer participation in the whole development process (Hamidan 2019; Pawar and Talekar 2023). Agricultural credit may take various forms, including seed loans, deferred payment fertilizer, the use of a tractor, labor, and storing facilities. In general, credit refers to the ability to borrow (Adewale et al. 2022). It addresses liquidity constraints faced by poor farmers, allowing them to purchase essential agricultural inputs such as fertilizers, seeds, pesticides, machinery, and other inputs associated with higher crop yields. Furthermore, agricultural credit catalyzes transforming the agriculture sector and drives the productivity of cereal crops (Musembi 2019). Access to credit also supports rural households to diversify their sources of income, expand their sources of capital, and control sudden shocks and stress (Ninh and Kieu 2019).

However, limited access to agricultural credit impedes the adoption of improved agricultural technologies in developing countries such as Ethiopia. Despite the increase in Micro‐Financial Institutions (MFIs), only 22% of adults in Ethiopia have access to financial services, compared with 29% in Sub‐Saharan Africa (SSA) and 62% globally (Demirgüç‐Kunt et al. 2015; Morduch 2010). This gap may arise from both supply‐side and demand‐side constraints. On the supply side, challenges include inadequate credit supply and high borrowing costs. On the demand side, factors such as risk aversion, high transaction costs, and limited information reduce farmers' willingness to seek credit. These barriers hinder rural households' use of agricultural credit, which in turn significantly affects the adoption of improved agricultural technologies (Balana et al. 2022). Despite low interest rates, farmers may still avoid taking credit due to collateral requirements, unaffordable repayment schedules, risk aversion, and the potential loss of collateral.

On the other hand, inflation deteriorates the purchasing power of the currency and increases the cost of agricultural inputs, including fertilizer, pesticide, seeds, and machinery. Such an economic phenomenon can lead to reduced agricultural output, influence food security, and economic stability (Ifeanyi Obeagu 2023; Mekonen 2020). Understanding the dynamic nature between inflation and CCP is very important for designing appropriate policies to support the agriculture sector and ensure food security in Ethiopia. Inflation rate of Ethiopia increased from 4.6% in 1980–1989 to 13.65% (2010–2018). Thus, the general price level has consistently exhibited a percentage increase (Mekonen 2020). As the agriculture sector was highly affected by inflation, it led to lower output levels and higher food prices (Nneoma 2024).

Theoretically, inflation has a direct relationship with agricultural output and economic growth (Danby 2005). However, empirical studies revealed that inflation and agricultural output had a negative correlation in developing countries. For instance, Mekonen (2020) investigates the impact of inflation using ARDL estimation techniques and reveals that inflation has a significant and adverse long‐run relationship with agriculture sector growth. The study by Janet (2024) investigates the impact of inflation on agricultural output in Nigeria and concludes that inflation has a significant negative impact on agricultural productivity. Similarly, Jame (2024) concluded that a high level of inflation significantly hinders agricultural output by raising production costs and diminishing farmers' purchasing power.

While global literature extensively documents the impact of climate change, agricultural credit, fertilizer price and subsidy on agricultural productivity (Ahsan et al. 2020; Kumar et al. 2021; Larik et al. 2024; Yovo 2023), Context‐specific studies in Ethiopia remain fragmented and inconclusive. In Ethiopia, existing studies are focused on the impact of climate variables, agricultural credit and subsidies on agricultural productivity using the ARDL model (Asfew and Bedemo 2022; Gebeyehu and Bedemo 2024; Ketema 2020) and using the production function and Ricardian approach (Bouteska et al. 2024). These studies ignore the potential impact of macroeconomic instability, such as inflation, on CCP. Moreover, most studies rely on static or traditional econometric techniques, which fail to capture dynamic adjustment paths.

As novelty, this study employed a Dynamic Simulated ARDL approach that captures the shock response of the independent variables on CCP in Ethiopia. This approach enables more accurate simulation of dynamic impacts compared to conventional ARDL models. Moreover, unlike previous studies that focus mainly on climatic variables, this study integrates agricultural credit and inflation into the productivity–climate nexus. Furthermore, the analysis is conducted in the Ethiopian context, where such a comprehensive framework has not been previously explored.

This knowledge gap hampers the design and implementation of macroeconomic policy on the provision of agricultural credit and on mitigating the adverse impact of inflation on CCPs. Consequently, this study was conducted to address the identified gap by answering the following two key questions: (1) How do climate change, agricultural credit, and inflation influence CCP in Ethiopia in the short and long run? and (2) How do CCP levels respond dynamically to shocks in climate variables, agricultural credit, and inflation? Understanding the combined effects of climate change, credit availability, and inflation is crucial for Ethiopia, where agriculture remains the backbone of the economy and is highly vulnerable to climate and economic shocks. Policymakers also require dynamic evidence to design effective climate‐resilient and finance‐supported agricultural policies.

## 2 Research Framework and Theoretical Basis

### 2.1 Theoretical Foundations

Theoretically, a combination of economic, environmental, and policy‐related variables can shape the productivity of cereal crops in developing countries like Ethiopia. As a result, the conceptual foundation of this study draws from 4 major theoretical streams, including climate change Ricardian theory, agricultural production and input theory, credit constraint theory, and cost‐push inflation theory.

#### 2.1.1 Climate Change (Ricardian) Theory

The Ricardian climate change impact theory posits that variations in carbon dioxide (CO_2_) emission, temperature and rainfall directly influence agricultural productivity through changes in crop growth cycles, evapotranspiration rates, soil moisture levels, and the frequency of extreme weather events (Kelly and Adger 2000; Lobell and Gourdji 2012; Mendelsohn et al. 1994; Mendelsohn and Dinar 2009). Climate change also hampers crop production by altering pest incidence and plant‐pest interaction (Juroszek and von Tiedemann 2013). For rain‐fed agricultural systems such as those dominant in Ethiopia, temperature anomalies and rainfall variability are key determinants of cereal crop yield outcomes. These theories support the inclusion of climate variables as fundamental drivers of productivity (Habtemariam et al. 2017).

#### 2.1.2 Credit Constraint Theory

Credit constraint theory argues that access to agricultural credit for smallholder and commercial farmers plays a significant role in alleviating financial constraints, enabling them to purchase and adopt productivity‐enhancing inputs such as improved seeds, fertilizers, and pesticides. This theory suggests that financial development supports agricultural productivity (Claessens and Erik 2007). On the contrary, limited and unaffordable agricultural credit service from the supply side forces farmers to under‐invest, resulting in lower agricultural yields (Ahmad 2011; Boansi et al. 2024; Guirkinger and Boucher 2008; Kinuthia 2018). In addition, credit constraints occur when lenders set high collateral requirements or strong contracts to secure their money back, and as a result, borrowers end up facing limits on how much credit they can access. These financial barriers shape borrowers' choice on how much they invest, the input they can afford and how they manage their credit over time (Kiyotaki and Moore 1997). This theory supports the expected positive role of agricultural credit on CCP in Ethiopia.

#### 2.1.3 Cost‐Push Inflation Theory

Macroeconomic price theory suggests that when inflation is high, the cost of agricultural inputs like seeds, fertilizer, fuel, and equipment becomes more expensive. According to Johnson (1980), inflation hurts agriculture more than most other sectors because it raises the cost of farm inputs, disrupts how resources are used, and often leads to policy responses that make the situation ever worse. Aye and Odhiambo (2021) state that the inflation threshold in a developing country is 5.997% and above this threshold, inflation has a negative effect on agricultural growth. Thus, inflation is theorized to have a negative impact on CCP due to higher production costs and reduced affordability of essential inputs.

#### 2.1.4 Practices of Agricultural Subsidy: Production Support and Market‐Failure Theories

Agricultural subsidies have a dual theoretical interpretation. On one hand, subsidies in developing countries help small farmers to afford essential inputs like fertilizer and improved seeds, so that they can overcome market imperfections; agriculture becomes more affordable and less risky (Chirwa and Dorward 2013; Hemming et al. 2018). On the other hand, classical economic theory suggests that subsidies can also reduce efficiency, distort the market, and create dependency if not properly managed. Inflation also affects the performance of crop productivity in developing countries (Eland 1985). The cost–price squeeze theory suggests that when the price of general goods and services rises, so does the price of farm inputs (Moss 1992). If the cost of agricultural input is higher than the output price, thereby eroding the profitability of the farm and discourages smallholder farmers from engaging in the agricultural sector.

#### 2.1.5 Agricultural Production and Input Theory

Agricultural production theory explains that total output is the function of inputs and it postulates how farmers convert inputs into outputs (Beattie et al. 2009; Debertin 2012). Inputs such as pesticides, fertilizers, cropland, and subsidies influence crop yield through their effects on soil fertility, pest resistance, and production efficiency (Jayne and Rashid 2013; Shah and Wu 2019). These relationships provide a theoretical basis for assessing how input intensity affects cereal productivity in Ethiopia. According to Cobb and Douglas, the three‐factor relationship exists among land, capital, and output (Cobb and Douglas 1928), and mathematically it can be expressed as follows:

$$\text{Output}=f\left(\text{land},\text{capital}\right)$$

This theory supports the expected positive role of agricultural input on CCP in Ethiopia.

### 2.2 Conceptual Framework

**FIGURE 1:**
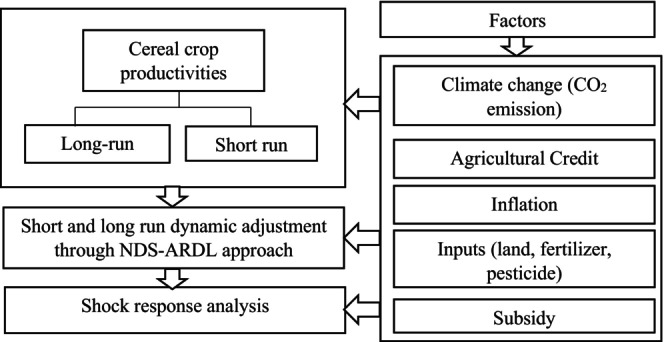
Conceptual framework of the study.

Based on an integrated theoretical foundation drawing on Ricardian climate change impact theory, agricultural production theory, and the agricultural finance literature, this study develops a comprehensive conceptual framework to explain cereal CCP in Ethiopia (Figure 1). The framework posits that climatic, financial, macroeconomic, and input‐related factors jointly determine the productivity of cereal crops. In line with Ricardian theory, climate change affects agricultural productivity by altering temperature, rainfall, and extreme weather patterns, which directly influence crop growth and yields (Mendelsohn and Dinar 2009; Ricardo 2005). Accordingly, the framework incorporates agricultural credit as a critical enabling factor that enhances farmers' adaptive capacity by facilitating the adoption of improved agricultural practices (Feder et al. 1990). Access to credit is therefore hypothesized to have a positive effect, mitigating the adverse impacts of climate change through improved input utilization. The framework further integrates macroeconomic instability, represented by inflation, as a structural constraint affecting agricultural productivity. High inflation erodes the real value of agricultural credit, increases input prices, and reduces farmers' ability to invest optimally in productivity‐enhancing technologies (Mundlak 2001). Inflation is thus expected to negatively influence CCP and to weaken the effectiveness of credit and input use. Finally, agricultural input utilization serves as a key transmission channel through which climate conditions, credit availability, and macroeconomic factors affect productivity. Consistent with agricultural production theory, the efficient use of inputs such as fertilizers, cropland, and pesticides is central to increasing crop yields (Sahota 1973).

### 2.3 Hypothesis of the Study

Based on the theory and conceptual framework guiding this study, the following hypothesis is forwarded:

- **H1:** Climate change proxy to CO_2_ emission has a negative and significant effect on CCP.
- **H2:** Agricultural credit has a positive and significant effect on CCP.
- **H3:** Inflation negatively influences CCP.
- **H4:** Agricultural inputs (cropland, fertilizer, pesticide) positively impact CCP.
- **H5:** Agricultural subsidy has a positive and significant impact on CCP.
- **H6:** Shocks to climate variables (CO_2_ emission), agricultural credit, inflation, agricultural inputs (cropland, fertilizer, pesticide) and subsidies generate measurable dynamic responses in CCP.

## 3 Methodology

### 3.1 Data Source and Description of Data

The study used annual time‐series data from 1992 to 2022 for Ethiopia. The data for Ethiopia is collected from Food and Agriculture Organization (FAO) and World Development Indicators (WDIs). EViews 12 and Stata 17 software were used to analyze the data. EViews 12 was employed for time‐series estimation, model testing, and ARDL‐based analysis, while Stata 17 was particularly used for shock response simulation and dynamic visualization of counterfactual scenarios in NDS‐ARDL framework.

**FIGURE 2:**
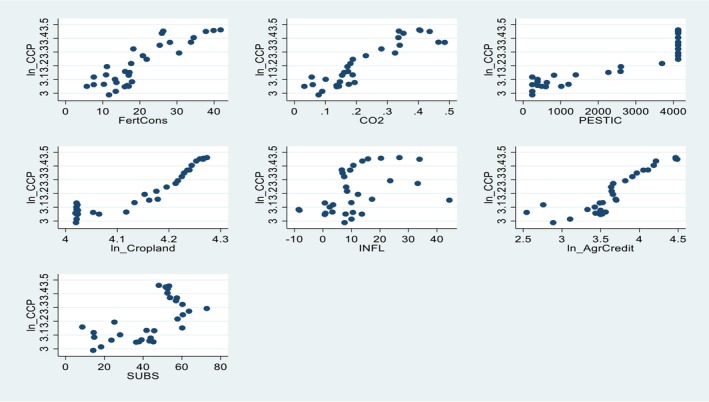
Plots of time series data.

Table 1 indicates the source and measurement units of the variables considered in this study. CCP was used as the dependent variable, whereas climate change (CO_2_ emission), agricultural credit, and inflation rate were the main explanatory variables in CCP. In addition, cropland, pesticide use, and agricultural subsidy were used as control variables. A 31‐year time series, spanning from 1992 to 2022, is used for both the explanatory and dependent variables. The descriptive statistics from Table 1 were supported by the scatter plot shown in Figure 2.

**TABLE 1:** Description of the variable and source of data.

Variables	Unit	Source	Obs	Mean	Std. dev.
Cereal crop productivity (CCP)	Kg/ha	WDIs	31	1739.158	645.638
Agricultural credit	Mill USD	FAO	31	8387.841	9280.866
Fertilizer Consumption	Kg/ha	WDIs	31	20.185	9.863
Carbon dioxide (CO_2_) emissions from Agriculture	Mt Co_2_e	WDIs	31	0.224	0.129
Pesticides (estimated total) for agriculture use	Ton	FAO	31	2358.721	1714.784
Cropland	Ha	WDIs	31	14181.87	3149.898
Inflation, consumer prices	% (annual)	FAO	31	11.415	11.611
Subsidies and other transfers	% (expense)	WDIs	31	43.063	17.0196

### 3.2 Model Specification Procedure

The use of appropriate estimation techniques for collected empirical data is important to evaluate the impact of climate change, agricultural credit and inflation on the productivity of cereal crops in Ethiopia. The ARDL model of Pesaran et al. (2001) was employed in this empirical study to conduct the bounds cointegration tests, which determine whether a long‐run relationship exists among variables under investigation. In addition, compared to other conventional cointegration methods like the Engle and Granger model (Engle and Granger 1987) or Johansen (Johansen 1992), the ARDL model is preferred due to the following arguments. Unlike the earlier methodologies, it can be used to analyze a dataset with a small sample size (Duasa 2007). Additionally, the ARDL approach is highly flexible in regressing variable integration orders (Nasrullah et al. 2021) and can estimate long‐ and short‐run cointegration, whether variables are I (0), I (1), or a mix of both (Frimpong and Oteng‐Abayie 2006; Pesaran et al. 2001; Shahbaz et al. 2013). It also allows simultaneous regression of long‐run and short‐run cointegration (Anh et al. 2020; Warsame et al. 2021). It estimates the error correction coefficient to adjust the short‐run equilibrium to the long‐run equilibrium. Techniques like bias‐corrected bootstrap are employed to improve the accuracy and reliability of statistical inference regarding long‐run cointegration among interested variables being considered, which in turn ensure more robust results (Warsame et al. 2022). The economic function of our study is shown in Equation (1) as follows:

(1) $$\begin{array}{c} {\mathrm{CCP}}_{t}=\alpha +\text{FertCons}+{\mathrm{Co}}_{2}+\text{PESTIC}+\text{INFL}\\ +\text{AgCred}+\text{SUBS}+\text{Cropland} \end{array}$$

${\mathrm{CCP}}_{t}$ Stands for CCP, FertCons stands for fertilizer consumption, Co_2_ stands for carbon dioxide emission, PESTIC stands for pesticide applied, INFL stands for inflation at constant price in percent, AgCred stands for provision of agricultural credit, SUBS stands for agricultural subsidy for key agricultural inputs and Cropland stands for arable land allocated for crop production.

We transformed Equation (1) into a log‐linear form, the natural logarithm is applied, as illustrated in Equation (2), to resolve the issue of heteroscedasticity, where all the variables and symbols remain the same as in Equation (1):

(2) $$\begin{array}{c} \ln\_{\mathrm{CCP}}_{t}=\beta_{0}+\beta_{1}\ln\_\text{FertCons}+\beta_{2}\ln\_{\mathrm{Co}}_{2}\\ +\beta_{3}\ln\_\text{PESTIC}+\beta_{4}\ln\_\text{INFL}+\beta_{5}\ln\_\text{AgCred}\\ +\beta_{6}\ln\_\text{SUBS}+\beta_{7}\ln\_\text{Cropland}+\mu_{t} \end{array}$$

The *ln* illustrates the logarithmic form, while *t* represents time and *μ* is the random error term. The coefficients of the regressors are depicted by β_1,2,3,4,5,6_,_7_.

### 3.3 Unit Root Test

Primarily, the selection of the model for time series analysis is guided by the result of the unit root test, which determines whether the variables are stationary or not at level, first difference, or both (Udoh 2011). Stationarity can be fulfilled if the mean, variance, and covariance are constant and do not vary with time. The Augmented Dickey‐Fuller (ADF) test was developed by Dickey and Fuller (1979), and the Phillips‐Perron (PP) test proposed by Phillips and Perron (1988). In this study, we used the Phillips‐Perron (PP) test in which the null hypothesis (H0) suggests that the series has a unit root (i.e., it is non‐stationary).

(3) $$∆Z_{t}=\;ɸ+p*Z_{t-1}++\varepsilon_{i}$$

where $Z_{t}$ is stands for time series. ɸ is the noise for hypothesis, *p* is the optimum number of lags for CCPs and $\varepsilon_{i}$ is the untainted white noise error term.

#### 3.3.1 Short‐Run and Long‐Run ARDL Model

The ARDL estimation method examines the long‐run and short‐run correlation among the variables under investigation. The model is defined as follows:

(4) $$\begin{array}{c} ∆{\text{CerPro}}_{t}=\;\alpha_{0}+\sum_{i=1}^{p}a_{1i}∆{\text{CerPro}}_{t-1}+\sum_{i=0}^{q}a_{2i}∆{\text{AgCred}}_{t-1}\\ +\sum_{i=0}^{q}a_{3i}∆{\text{FertCons}}_{t-1}+\sum_{i=0}^{q}a_{4i}∆\mathrm{CO}2_{t-1}+\sum_{i=0}^{q}a_{5i}∆{\text{PESTIC}}_{t-1}\\ +\sum_{i=0}^{q}a_{6i}∆{\text{CroLND}}_{t-1}+\sum_{i=0}^{q}a_{7i}∆{\text{INFL}}_{t-1}+\sum_{i=0}^{q}a_{8i}∆{\text{SUBS}}_{t-1}\\ +b_{1}{\text{CerPro}}_{t-1}+b_{2}{\text{AgCred}}_{t-1}+b_{3}{\text{FertCons}}_{t-1}+b_{4}\mathrm{CO}2_{t-1}\\ +b_{5}{\text{PESTIC}}_{t-1}+b_{6}{\text{CroLND}}_{t-1}+b_{1}{\text{INFL}}_{t-1}+b_{1}{\text{SUBS}}_{t-1}+\varepsilon_{t} \end{array}$$

∆ represent first difference; 𝑎𝑜, constant; short‐term effects; ai stands for short‐term coefficients, 𝑏𝑖 stands for long‐term dynamic interaction of the model; $\varepsilon_{t}$, error term (white noise).

#### 3.3.2 Bound Test

From the very beginning, the ARDL model starts its job by testing the existence of cointegration among the variables considered in the study (Frimpong and Oteng‐Abayie 2006). Quite a few studies carried out several cointegration tests, including Engle and Granger (1987), Johansen (1991), Johansen and Juselius (1990), and Pesaran et al. (2001). Engle and Granger (1987) cointegration test is used for only two integrated variables of the same order by neglecting different mixed orders of integration (Kuma 2018). Johansen's test solves this drawback using error‐correction autoregressive vector modeling (VECM). When variables exhibit different integration orders (I (0) and I (1)), the cointegration test developed by Pesaran et al. (2001), known as the “bounds test for cointegration,” can be utilized.

The long‐term relationship between variables could be checked by carrying out a cointegration test. If the calculated *F*‐statistic is lower than the lower bound, the researcher is failing to reject the null hypothesis of no long‐run cointegration. If the *F*‐statistic falls between the lower limit and the upper limit, the result is indecisive, meaning no definitive conclusion can be made about the long‐term relationship. However, if the *F*‐statistic exceeds the upper bound, the researcher is failing to accept the null hypothesis of no cointegration, confirming the presence of a long‐term relationship between variables. The null and alternative hypotheses tested are as follows:

$$\begin{array}{c} \text{H0}:\text{b1}i=\text{b2}i=\text{b3}i=\text{b4}i=\text{b5}i=\text{b6}i=\text{b7}i=\text{b8}i:\\ \text{Existence of}\;a\;\text{cointegrating relationship}. \end{array}$$

$$\begin{array}{c} \text{H1}:\text{b1}i\neq \text{b2}i\neq \text{b3}i\neq \text{b4}i\neq \text{b5}i\neq \text{b6}i\neq \text{b7}i\neq \text{b8}i:\\ \text{Absence of}\;a\;\text{cointegrating relationship}. \end{array}$$

As with any dynamic model, the Akaikian information criteria (Akaike‐AIC, Shwarz‐SIC) are used to determine the optimal offsets (p, q) of the ARDL model.

Once we ensure the existence of a long‐term cointegrating, an error‐correction model can be used to verify the presence of cointegration between variables. The model is specified as follows:

(5) $$\begin{array}{c} ∆{\text{CerPro}}_{t}=\alpha_{0}+\sum_{i=1}^{p}a_{1i}∆{\text{CerPro}}_{t-1}+\sum_{i=0}^{q}a_{2i}∆{\text{AgCred}}_{t-1}\\ +\sum_{i=0}^{q}a_{3i}∆{\text{FertCons}}_{t-1}+\sum_{i=0}^{q}a_{4i}∆\mathrm{CO}2_{t-1}+\sum_{i=0}^{q}a_{5i}∆{\text{PESTIC}}_{t-1}\\ +\sum_{i=0}^{q}a_{6i}∆{\text{CroLND}}_{t-1}+\sum_{i=0}^{q}a_{7i}∆{\text{INFL}}_{t-1}+\sum_{i=0}^{q}a_{8i}∆{\text{SUBS}}_{t-1}\\ +\theta {\mathrm{ECM}}_{t-1}+\varepsilon_{t} \end{array}$$

where θ represents the speed of adjustment. A negative and statistically significant error correction term (ECT) of less than 1 reveals that the variables will adjust back to equilibrium in events of any deviation from the long run. On the other hand, a positive ECT implies the variable may not converge to its equilibrium point.

### 3.4 Novel Dynamic Simulated ARDL Estimation Techniques

Even though the ARDL model is relatively straightforward, its dynamic structure, incorporating various lags including first difference and lagged difference, can complicate the economic interpretation of regressors' effects (AlNemer et al. 2023; Sarkodie and Owusu 2020). To address this, Jordan and Philips (Jordan and Philips 2018) introduced the Novel Dynamic Simulated‐ARDL (NDS‐ARDL) techniques. The NDS‐ARDL is an extension of the standard ARDL approach by allowing explanatory variables to respond asymmetrically and dynamically to positive and negative shocks (Ghose et al. 2024). Unlike the conventional ARDL model, which assumes linear and symmetric adjustments, the NDS‐ARDL model separately estimates the impact of upward and downward changes in each independent variable. Advantageously, the NDS‐ARDL model includes a visual interface that allows the researcher to explore how a 10 unit counterfactual shock in a selected variable would affect the CCP, at ceteris paribus (Sarkodie and Owusu 2020). By capturing the fact that a change in the same variable may not produce identical effects, the NDS‐ARDL provides a more flexible and realistic representation of both short‐ and long‐run dynamics, particularly in the context where asymmetric responses are important.

Accordingly, the NDS‐ARDL model is specified in the following equations.

(6) $$\begin{array}{c} ∆\ln\_{\mathrm{CCP}}_{t}=\gamma_{0}+\beta_{0}\ln\_{\mathrm{CCP}}_{t-1}+\beta_{1}\ln\_{\text{FertCons}}_{t}\\ +\beta_{1}∆\ln\_{\text{FertCons}}_{t-1}+\beta_{2}\ln\_{{\mathrm{Co}}_{2}}_{t}+\beta_{2}∆\ln\_{{\mathrm{Co}}_{2}}_{t-1}\\ +\beta_{3}\ln\_{\text{PESTIC}}_{t}+\beta_{3}∆\ln\_{\text{PESTIC}}_{t-1}+\beta_{4}\ln\_{\text{INFL}}_{t}\\ +\beta_{4}∆\ln\_{\text{INFL}}_{t-1}+\beta_{5}\ln\_{\text{AgCred}}_{t}+\beta_{5}∆\ln\_{\text{AgCred}}_{t-1}\\ +\beta_{6}\ln\_{\text{SUBS}}_{t}+\beta_{6}∆\ln\_{\text{SUBS}}_{t-1}+\beta_{7}\ln\_{\text{Cropland}}_{t}\\ +\beta_{7}∆\ln\_{\text{Cropland}}_{t-1}+\mu_{t} \end{array}$$

### 3.5 Methodological Flowchart

**FIGURE 3:**
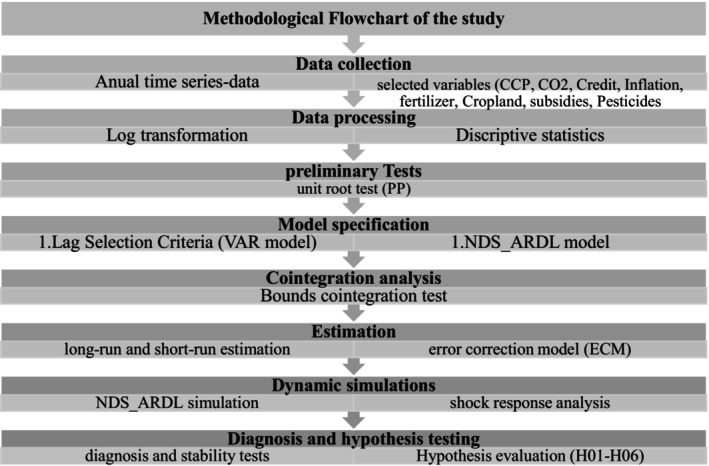
Methodological flowchart.

Figure 3 presents a methodological flowchart summarizing the sequential steps followed in this study, from data collection and preprocessing to model estimation, diagnostic testing, and dynamic simulation analysis.

## 4 Results

### 4.1 Unit Root Test

Ensuring the stationarity of the variable in time series data analysis is the fundamental job of the researcher. To determine the order of integration (OI) in the levels I (0) and first difference I (1) for the selected variables, this study employed Phillips‐Perron unit root test. The test result revealed that initially, all variables were not stationary at the level I (0) and became stationary after first differencing I (1) according to the Phillips‐Perron test statistic value. The results of the Phillips‐Perron test are shown in Table 2.

**TABLE 2:** Result of Phillips‐Perron test statistic (PP test) stationarity tests.

Variables	PP at I (0)		PP at I (1)	
	*t*‐statistics	*p*	*t*‐statistics	*p*
LnCCP	0.490	0.984	−6.844	0.000
lnCo2	−0.723	0.825	−8.515	0.000
lnFertCons	−1.061	0.000	−12.823	0.000
INFL	–2.916	0.060	−9.522	0.000
lnLn_AgrCred	−1.947	0.307	−3.957	0.005
lnLn_Croland	−0.167	0.932	−4.061	0.003
lnPESTIC	−1.658	0.441	−5.216	0.000
SUBS	−2.077	0.254	−6.663	0.000

### 4.2 Lag Selection Criteria (VAR Model)

To conduct NDS‐ARDL bound test, choosing an appropriate lag order is a prime statistical procedure to ascertain whether cointegration exists between CCP, agricultural credit, fertilizer consumption, climate change (Co_2_), Cropland, pesticide, inflation and subsidy. To do so, this investigation adopts VAR model's optimal lag order, and the results are illustrated in Table 3. The most popular AIC and SIC lag selection criteria were used to determine the lag order under this investigation. As AIC results revealed, lag 2 is the appropriate lag order for the variables considered in this study.

**TABLE 3:** Lag selection criteria.

Lag	LogL	LR	FPE	AIC	SC	HQ
0	−362.6487	NA	17.44723	25.56198	25.93916	25.68011
1	−198.0357	227.0524	0.020029	18.62315	22.01782	19.68632
2	−78.05549	99.29396^a^	0.001402^a^	14.76245^a^	21.17459^a^	16.77065^a^

^a^ The optimal lag length selected by each criterion. Specifically, it marks the lag order at which the value of the respective information criterion (LR, FPE, AIC, SC, and HQ) is minimized.

### 4.3 Bounds Cointegration Test

After the static ARDL model estimation, the results of the bounds cointegration tests indicate the presence of a cointegration relationship between the series under investigation. The test result confirmed a long‐term relationship at a 1% significance level between the dependent variable, CCP, and the other variables under investigation. Table 4 revealed that the F calculated statistic (7.165991) exceeds the upper bound critical value (3.21) at the 5% threshold. This suggests that CCP and its determinants, such as agricultural credit, Co_2_, fertilizer consumption, crop land, pesticide inflation and agricultural subsidy, have a strong long‐term relationship. Thus, the researchers are failing to accept the null hypotheses of no cointegration, which verify the existence of long‐term cointegrating relationships among the series.

**TABLE 4:** Results of the cointegration test.

Variables	Ln_CCP ln_AgrCred Co_2_ FertCons Cropland PESTIC INFL SUBS	
*F*‐Statistics	7.165991	
Critical threshold	Lower bound I (o)	Upper bound I (1)
1%	2.73	3.9
2.5%	2.43	3.51
5%	2.17	3.21
10%	1.92	2.89

### 4.4 Novel Dynamic Simulated ARDL Model Estimation Result: Long‐Run and Short‐Run

Table 5 represents the estimated regression coefficient derived from the NDS‐ARDL model. The results show that the estimated error correction term (ECT) is negative (−0.674) and highly significant, verifying the presence of a long‐run cointegration relationship.

**TABLE 5:** NDS‐ARDL long and short‐run estimation.

CCP	Coefficient	Std. err.	*T*	*P*>t
lnCCP	−0.7985328	0.1260265	−6.34	0.000
**Long‐run estimation**				
lnCo2	−0.378	0.169	−2.23	0.042
lnFertCons	0.005	0.002	2.50	0.025
lnPESTIC	−0.00003	0.0000203	−1.48	0.161
lnCropland	1.641	0.415	3.95	0.001
INFL	−0.004	0.001	−3.86	0.002
lnAgrCred	0.065	0.0385441	1.68	0.114
SUBS	0.001	0.0007	2.19	0.046
**Short‐run estimation**				
_cons	−4.430	1.573	−2.82	0.014
∆ln CO2	−0.108	0.1246	−0.87	0.398
∆ln FertCons	0.002	0.001	1.29	0.216
∆ln PESTIC	0.0000209	0.00002	0.98	0.345
∆ln Cropland	−0.871	0.527	−1.65	0.121
∆ INFL	−0.002	0.0007	−2.19	0.046
∆ln AgrCred	0.216	0.077	2.78	0.015
∆ SUBS	0.0001	0.0006	0.16	0.878
ECT_CointEq (−1)*	−0.674	0.064	−10.55455	0.000
*F* (15, 14) =	5.38	Prob >F	=0.0016	
*R*‐squared	0.852	Adj *R*‐squared	=0.6936	
Simulation	1000	Observation	30	

In the long run, CO_2_ emissions, fertilizer consumption, cropland, inflation, and subsidy have a statistically significant impact on CCP in Ethiopia, while in the short run, agricultural credit and inflation are the significant determinants. The long‐run estimation results revealed that CO2 emissions have a statistically significant negative impact on CCP at the 5% level. Specifically, a 1% increase in CO_2_ emissions (in mt) is associated with about a 38% reduction in crop productivity. Conversely, fertilizer consumption has a significant and positive impact on CCP in the long run, with a 1% increase in fertilizer use (kg/ha) led to a 5% improvement in crop productivity.

Moreover, cropland shows a significant and positive association with crop productivity at the 1% level, implying that a 1% increase in cropland area leads to a 1.64% rise in crop productivity in the long run. The long‐run estimates also reveal that agricultural subsidies have a positive and significant effect on crop productivity. A 1% increase in agricultural subsidies would increase the productivity of cereal crops by 1%. In addition, the model's results uncover that inflation has a significant negative impact on CCP, with a 1% rise in inflation reducing productivity by about 4% in the long run and 2% in the short run.

The short‐run estimate of the model stated that the provision of agricultural credit has a positive and significant effect on crop productivity at the 5% level. Finally, the model shows that pesticide use does not have a statistically significant relationship with crop productivity in either the long run or the short run.

### 4.5 Shock (Impulse) Response Analysis of Novel Dynamic ARDL Simulation

**FIGURE 4:**
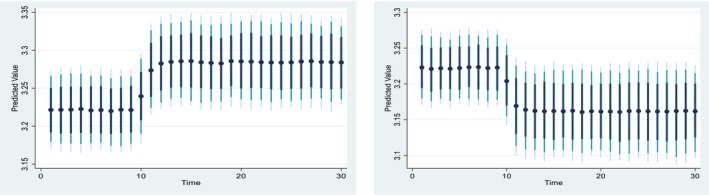
Change (±10) fertilizer consumption and its effect on Ln‐cereal yield.

**FIGURE 5:**
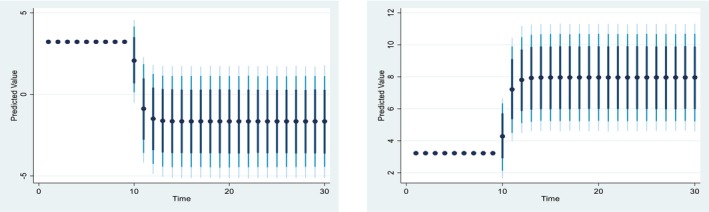
Change (±10) CO_2_ and its effect on Ln‐cereal yield.

**FIGURE 6:**
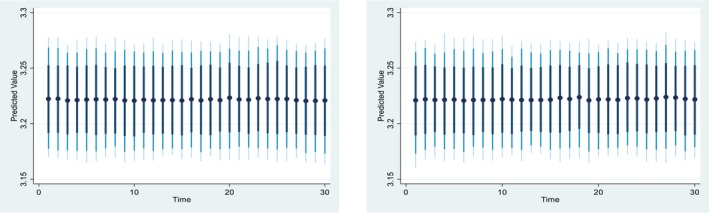
Change (±10) pesticide and its effect on Ln‐cereal yield.

**FIGURE 7:**
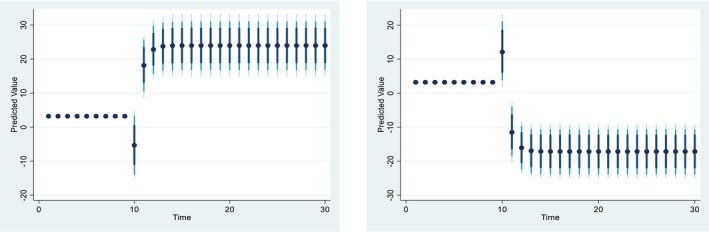
Change (±10) ln_Cropland and its effect on Ln‐cereal yield.

**FIGURE 8:**
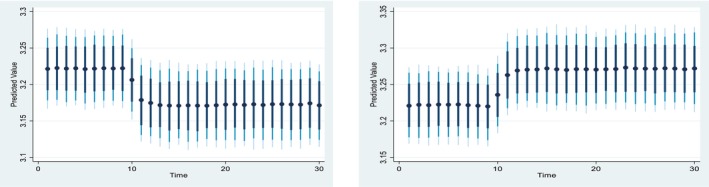
Change (±10) inflation and its effect on Ln‐cereal yield.

**FIGURE 9:**
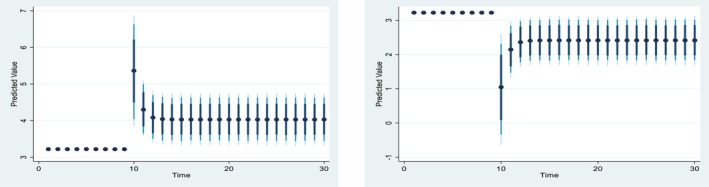
Change (±10) ln‐agrcredit and its effect on Ln‐CCP.

**FIGURE 10:**
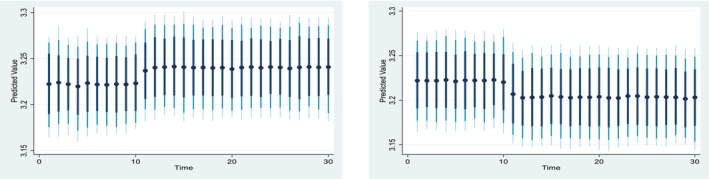
Change (±10) subsidy and its effect on Ln‐CCP.

Figures 4, 5, 6, 7, 8, 9, 10 presents the NDS‐ARDL dynamic simulation plots, generated from 1000 simulations across 30 time points, with 10 positive and 10 negative shocks to each regressor. The resulting counterfactual response of the dependent variable generally supports Hypothesis H6, which concerns changes in the dependent variable. All shock responses support Hypothesis H6, except for the dynamics observed in Figure 6.

Figure 4 illustrates the impact of a ±10% shock in fertilizer application on CCP. A 10% increase in fertilizer consumption leads to a rise in CCP in the long run, whereas a 10% decrease in predicted fertilizer consumption results in a sharper decline in CCP. Both the ARDL and NDS‐ARDL results are consistent, confirming that the long‐run effects of fertilizer shocks on CCP are more significant than those in the short run.

Figure 5 is based on 10% positive and negative CO2 shocks to CCP. A 10% positive shock from climate change (CO2) would significantly reduce CCP from around 3% to around −2% in the long run. In contrast, a 10% negative shock from CO2 would increase CCP from approximately 3% to 8%. Overall, the effect of a CO2 shock is more significant in the long run than in the short run.

Figure 6 indicates the impact of ±10 shocks in predicted pesticide use on CCP, indicating that CCP remains largely unchanged following both positive and negative shocks in predicted pesticide use. Turning to cropland, Figure 7 indicates the impact of ±10 shocks in predicted cropland on CCP. Specifically, a 10% increase in cropland leads to a rise in CCP in the long run, whereas a 10% decrease in cropland results in a steady decline in CCP. Overall, the effects of cropland shocks on CCP differ markedly between positive and negative changes and across the short and long run.

Figure 8 presented 10% positive and negative shocks of inflation on CCP. A 10% increase in inflation slightly reduces CCP from around 3.225% to 3.175% in the long‐run and short‐run. On the other hand, a 10% negative shock from inflation leads to an improvement in CCP. Overall, the effect of the shock from the inflation is almost similar in the long‐run and the short run.

Figure 9 illustrate 10% positive and negative shocks to agricultural credit on CCP. A 10% increase in agricultural credit would significantly improve CCP in the short run, while a 10% negative shock to agricultural credit would lead to a reduction in CCP. On the other hand, Figure 10 shows the impact of 10% positive and negative shocks to predicted agricultural subsidies on CCP. A +10% shock to the predicted agricultural subsidy significantly improves CCP in the long run, whereas a −10% shock to the agricultural subsidy reduces CCP in the long run.

### 4.6 Diagnostic Testing

#### 4.6.1 Normality Test

The Jarque‐Bera test was employed to check the normality of the estimated residual error term (Table 6). From Table 6, the ARDL model is well behaved and normal since the probability value (*p*‐value) is insignificant, which indicates that we failed to reject the null hypothesis of residuals being normally distributed.

**TABLE 6:** Diagnostic test.

Diagnosis	Test	*F*‐statistics	Chi‐squared *p*
Normality test	Jarque‐Bera	4.496186	10.5600
Serial autocorrelation	LM test	1.024242	0.3974
Heteroscedasticity test	Breusch‐Pagan‐Godfrey test	0.737622	0.7226

#### 4.6.2 Serial Correlation Test (LM Test)

The LM Test for Breusch‐Godfrey Serial Correlation indicates a statistic of 1.024242 with a corresponding probability of 0.3974, verifying that there is no serial correlation in the model (Table 6).

#### 4.6.3 Heteroscedasticity Test

The Breusch‐Pagan‐Godfrey test was applied to investigate the heteroscedasticity problem in the data test. Hence, the *p*‐value of the *F*‐statistic is 0.7226, which is > 5%, the researcher fails to reject the null hypothesis and concludes there is no series heteroscedasticity problem in the data set (Table 6).

#### 4.6.4 Stability Test

**FIGURE 11:**
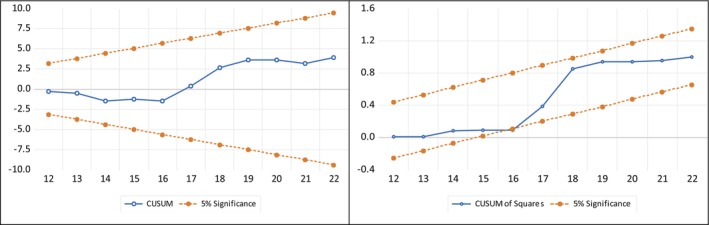
Stability test.

The Researchers conducted CUSUM (Cumulative Sum) and CUSUM of Squares tests for structural breaks in the long‐run equations to assess the stability and reliability of the fitted ARDL model. As illustrated in Figure 11, the result indicates no evidence of structural breaks, as the CUSUM and CUSUM of Squares test statistics for the error term remain within the two critical bounds (represented by the red line). It confirms that the parameters of the model are stable and robust over time, as depicted in Figure 11.

## 5 Discussion

### 5.1 Result Interpretation and Comparison With Previous Studies

The finding that the lagged value of CCP (lnCCP) has a negative and significant coefficient reveals strong mean reversion in CCP (Table 5). It indicates a self‐correcting dynamic in which deviations from long‐run productivity trends are partially reversed in subsequent periods. This behavior coincides with the theory of resilience in agriculture (Zampieri et al. 2019), which suggests that farmers respond to negative shocks by reallocating cropland and increasing the use of agricultural inputs such as fertilizer, pesticide, and improved seeds that help restore production levels. This study may provide new evidence that Ethiopia's CCP inherently adjusts itself in the long run after experiencing shocks. The existing literature in Ethiopia depicts the long‐run production trends, input use, and climate change impacts, while very few studies indicate the resilience behavior of cereal production using time series simulation methods. The finding is in line with (Agnolucci and De Lipsis 2020).

The negative and significant association between CO_2_ emission and CCP in the long‐run revealed that climate‐driven environmental change declines CCP over time in Ethiopia. This finding is consistent with recent empirical studies reporting adverse effects of emissions on agricultural performance in developing and climate‐vulnerable economies (Chandio et al. 2022; Khalifa 2025). This result is consistent with Climate Change (Ricardian) Theory and broader climate‐impact assessment, indicating that warming and altered precipitation patterns reduce crop productivity in Africa (Christopher et al. 2022). In contrast, other studies found that CO_2_ emissions had a significant positive impact on crop productivity (Ahsan et al. 2020; Asfew and Bedemo 2022). In Ethiopian context, since smallholder farmers rely on rain‐fed agriculture, climate change has an immediate and direct implication on CCP and food security among poor farming households. Therefore, promoting climate‐smart technologies aligns with Ethiopia's CRGE goals and evidence on crop insurance, requiring sustained public–private efforts to reduce CO_2_ impacts on agriculture sector (World Bank 2020).

The long‐run positive relationship between fertilizer use and agricultural productivity indicates that fertilizer is a key input for smallholder farmers operating on nutrient‐deficient soil and degraded lands in Ethiopia. This finding is consistent with empirical evidence indicating that fertilizer use has a significant impact on agricultural productivity (Asfew and Bedemo 2022; Gul et al. 2022; Rehman et al. 2019) and aligns with production theory, which posits that yield‐enhancing inputs generate sustained productivity gains when applied consistently over time. As a result, the long‐run elasticity results confirm that farmers with consistent access to fertilizer could increase cereal productivity gradually and stabilize at a higher level over time. The policy implication is that ensuring a reliable and affordable fertilizer supply is essential for sustaining long‐term productivity growth and food security in Ethiopia.

In the long‐run, cropland has a strong and significant positive impact on the productivity of cereal crops. This finding is consistent with recent empirical evidence showing that expansion or effective utilization of cropland enhances agricultural productivity in developing economies (Ahsan et al. 2020; Asfew and Bedemo 2022; Khan et al. 2020; Yu et al. 2019). In the Ethiopian context, this result supports the argument that underutilized or low‐productivity land can generate sustained cereal yield gains when complemented with climate‐smart practices and appropriate agronomic inputs (Wakjira et al. 2024). From a production and land‐use theory perspective, the result suggests that cereal output is highly responsive to changes in cultivated area, particularly where land constraints and technological intensity remain binding. The policy implication is that productivity gains should focus not only on cropland expansion but also on improving land‐use efficiency through sustainable land management, climate‐smart agriculture, and investments that transform area expansion into long‐term productivity growth.

The results from the model also uncovered that inflation had a negative impact on CCP, which stemmed from increased production costs, causing supply chain interruptions and economic instability in both the long and short run. As rising inflation drives up the cost of production, it leads to constraining the productivity of cereal crops. This implied that inflation is a direct threat to food security and rural livelihood. This finding is consistent with recent empirical evidence (Befikadu 2024; Gebeyehu and Bedemo 2024; Larik et al. 2024). From a theoretical perspective, this result aligns with cost‐push inflation and credit‐constraint theories, which posit that rising prices reduce producers' real input use and investment capacity, thereby lowering output. To protect the agricultural sector from price volatility, policymakers should implement prudent monetary and fiscal policies, ensure adequate foreign currency for basic farm inputs, provide crop‐specific subsidies, and expand agricultural credit services, which are critical for sustaining cereal productivity under inflationary pressure.

Furthermore, the findings indicate a strong and positive short‐run relationship between provision of agricultural credit and CCP. This implied that agricultural credit could help farmers increase their productivity by allowing them to invest in new technologies and inputs, integrate farmers into the agricultural value chain, invest in climate‐smart agriculture and manage production costs more efficiently (Larik et al. 2024; Verkuil et al. 2024; Murungi et al. 2023). It implied that access to rural credit services leads to an immediate impact on booming crop productivity. From a theoretical perspective, this result aligns with credit constraints, which posit that relaxing liquidity constraints allows farmers to respond quickly to production opportunities and cost pressures. In developing countries like Ethiopia, it also has a tremendous effect on income generation, a source of employment, increase farmer purchasing power and ensures food security. The policy implication is that expanding inclusive rural financial services, strengthening agricultural credit institutions, and integrating credit with extension and value‐chain development are essential for sustaining short‐run productivity gains and supporting broader rural development outcomes.

Agricultural subsidy for cereal crop producers plays a significant role in stabilizing income, food security, and supporting the rural economy. In this study, it also has a significant positive impact on agricultural productivity in the long run. The possible reason in the Ethiopian context could be that government support through agricultural subsidy leads to a reduction in the cost of the key inputs, namely fertilizer, improved crop seed, machinery, and irrigation. As a result, it would increase total factor productivity, farmer purchasing power, and the use of agricultural inputs efficiently to enhance CCP. Our finding is aligned with those of (Biagini et al. 2022; Li et al. 2025; Ye et al. 2024). From a theoretical perspective, this result aligns with production support and market‐failure theories, which argue that subsidies can correct input market imperfections and relax financial constraints faced by smallholder farmers. Policy makers should make sure subsidy truly reaches the smallholder farmers who need it the most.

### 5.2 Hypothesis Evaluation (H1, H2, H3, H4, H5, H6)

The evaluation of the study's hypotheses (H1, H2, H3, H4, H5, H6) is based on the long‐run and short‐run results obtained from the NDS‐ARDL estimations, as well as the dynamic simulation analyses.

Hypothesis H1 *Postulates that climate change, proxied by CO*_*2*_ *emissions, has a significant impact on CCP. The long‐run results indicate a statistically significant negative relationship between CO*_*2*_ *emissions and CCP, confirming that rising emissions adversely affect agricultural performance. Accordingly*, *H1* *is supported*.

Hypothesis H2 *Asserts that agricultural credit positively influences CCP. The short‐run estimates reveal that agricultural credit has a positive and statistically significant effect on CCP at the 5% significance level. This finding suggests that access to credit enhances farmers’ ability to invest in productivity‐enhancing inputs in the short term. Therefore*, *H2* *is supported*.

Hypothesis H3 *Examines the effect of inflation on CCP. The results show that inflation has a significant negative effect on productivity in both the long and short runs. Specifically, increases in inflation reduce crop productivity by raising input costs and weakening purchasing power. Thus*, *H3* *is supported*.

Hypothesis H4 *Relates to the role of agricultural input utilization, including fertilizer use and cropland area. The long‐run results show that fertilizer consumption and cropland expansion have positive and statistically significant effects on CCP. These findings highlight the importance of input utilization in enhancing agricultural output, thereby supporting* *H4*.

Hypothesis H5 *Proposes that agricultural subsidies improve CCP. The empirical results indicate that agricultural subsidies have a positive and significant effect on productivity, with a proportional increase in subsidies leading to an increase in crop output. Hence*, *H5* *is supported*.

Hypothesis H6 *Concerns the dynamic responses of CCP to positive and negative shocks in the explanatory variables. The NDS‐ARDL dynamic simulation results (Figures* *4*, *5*, *6*, *7*, *8*, *9*, *10* *) show that the counterfactual shock responses generally align with the hypothesized relationships, supporting* *H6*, *with the exception of the dynamics observed in Figure* *6*.

Overall, the hypothesis evaluation confirms that climate change, financial access, macroeconomic conditions, and agricultural inputs jointly play a critical role in shaping CCP in Ethiopia.

### 5.3 Theoretical Implication

This study offers important theoretical implications for agricultural productivity and climate change research in developing economies. The long‐run negative effect of CO_2_ emissions extends Climate Change (Ricardian) Theory by showing that climate‐induced stress outweighs potential CO_2_ fertilization benefits in rain‐fed, low‐adaptive systems. The positive long‐run roles of fertilizer use, cropland expansion, and subsidies support production input theory, while the short‐run significance of agricultural credit confirms credit‐constraint theory. Additionally, the adverse effect of inflation aligns with cost‐push inflation theory, highlighting the role of macroeconomic instability in constraining productivity. Overall, the findings emphasize the context‐dependent nature of agricultural productivity dynamics.

## 6 Conclusion and Recommendations

This study addresses the short‐run and long‐run effects of macroeconomic and agricultural input variables on CCP in Ethiopia using NDS‐ARDL approach with time series data from 1992 to 2022. It also examines how CCP responds to positive and negative shocks of ±10% in each regressors. The results indicate that CO2 emissions, fertilizer consumption, cropland area, inflation, and agricultural subsidies have significant long‐term effects on crop productivity. In contrast, inflation and agricultural credit are the primary short‐run determinants. The long‐run estimate implied that both environmental pressure and economic factors play a sustained role in shaping CCP. This demands a practical policy that balances agricultural expansion and environmental sustainability by keeping a stable input market and targeting input subsidies. Whereas the short‐run result indicates that farmers' immediate production function is determined by average price change and access to agricultural credit underscore the importance of timely credit service and appropriate monetary policy to control inflation.

Based on the NDS‐ARD framework results, the following recommendations were provided:

To address the negative outcome of the CO_2_ emission on long‐term crop productivity, both public and private sectors need to actively engage in implementing the Climate Resilient Green Economy (CRGE) initiative, introduced by the current government of Ethiopia. This initiative aims to minimize the risk associated with climate change while promoting sustainability development. In addition, farmer extension education should be provided for farmers to create awareness on utilization of location‐specific extension recommended fertilizer in efficient and responsible manners to boost agricultural productivity in the long‐run. Furthermore, efforts should be focused on efficient and sustainable use of existing cropland, making newly cultivated land productive through proper land preparation, soil conservation, and investment in irrigation. To reduce the negative consequence of cropland expansion, implementing land use planning and monitoring is also important. Since inflation has long‐run and short‐run negative impacts on CCP, policymakers should prioritize macroeconomic stability to safeguard agricultural productivity through strengthening inflation‐targeting measures, improving monetary discipline, and stabilizing input markets so that farmers can access key agricultural inputs, including credit at predictable prices.

Simultaneously, agricultural credit enhances CCP in the short run. Financial providing institutions should focus on delivering short‐term financial service for those who need it most with lower interest rates, collateral requirements, and transparent lending procedures. Strengthening rural credit & saving institutions and promoting multipurpose farmer cooperatives can significantly reduce financial constraints of rural farmers. Finally, targeted agricultural subsidies should prioritize support for sustainable agricultural practices such as drought resistance, improved seeds, sustainable irrigation, and soil and water conservation measures so that long‐term CCP can be achieved.

## 7 Future Prospective

While this study investigates and provides valuable insights into the determinants of CCP in Ethiopia, several areas warrant further investigation. Future research could incorporate additional climate‐related variables including temperature, precipitation, and climate variability indices to capture a more comprehensive climate‐related risk. Taking and analyzing special data allows for area‐specific policy recommendations, as climate‐related factors and resource variability vary significantly across Ethiopia's agroecological zones. Furthermore, future research can focus on digital farming tools, market connectivity, and irrigation which may also influence cereal productivity.

## Author Contributions

All authors contributed to the study conception and design. Conceptualization, investigation, writing original draft, methodology, writing – review and editing, software, formal analysis, data collection and curation were performed by Prof. Jianmin Cao, Gizachew Wosene and Yuhan Pang. The first original draft, methodology and review and editing of the manuscript were also written by Mezgebu Aynalem and Arshad Ullah Jadoon. All authors read and approved the final manuscript.

## Disclosure

The authors have nothing to report.

## Ethics Statement

This pertains to the moral standards that the researcher adhered to during the study, ascertaining proper acknowledgment of data sources. All information obtained from other authors was duly recognized and appropriately cited.

## Conflicts of Interest

The authors declare no conflicts of interest.

## Data Availability

The data sets used to analyze for this study will be presented by authors upon request.
